# Chloroquine Analog Interaction with C2- and Iota-Toxin in Vitro and in Living Cells

**DOI:** 10.3390/toxins8080237

**Published:** 2016-08-10

**Authors:** Angelika Kronhardt, Christoph Beitzinger, Holger Barth, Roland Benz

**Affiliations:** 1 Rudolf Virchow Center, Research Center for Experimental Biomedicine, University of Würzburg, Versbacher Straße 9, 97078 Würzburg, Germany; ange.kronhardt@web.de (A.K.); christoph.beitzinger@web.de (C.B.); 2 Institute of Pharmacology and Toxicology, University of Ulm Medical Center, Albert-Einstein-Allee 11, 89081 Ulm, Germany; holger.barth@uni-ulm.de; 3 Department of Life Sciences and Chemistry, Jacobs-University Bremen, Campus-Ring 1, 28759 Bremen, Germany

**Keywords:** C2-toxin, iota-toxin, binding components, chloroquine, black lipid bilayer, aminoquinolinium salts

## Abstract

C2-toxin from *Clostridium botulinum* and Iota-toxin from *Clostridium perfringens* belong both to the binary A-B-type of toxins consisting of two separately secreted components, an enzymatic subunit A and a binding component B that facilitates the entry of the corresponding enzymatic subunit into the target cells. The enzymatic subunits are in both cases actin ADP-ribosyltransferases that modify R177 of globular actin finally leading to cell death. Following their binding to host cells’ receptors and internalization, the two binding components form heptameric channels in endosomal membranes which mediate the translocation of the enzymatic components Iota a and C2I from endosomes into the cytosol of the target cells. The binding components form ion-permeable channels in artificial and biological membranes. Chloroquine and related 4-aminoquinolines were able to block channel formation in vitro and intoxication of living cells. In this study, we extended our previous work to the use of different chloroquine analogs and demonstrate that positively charged aminoquinolinium salts are able to block channels formed in lipid bilayer membranes by the binding components of C2- and Iota-toxin. Similarly, these molecules protect cultured mammalian cells from intoxication with C2- and Iota-toxin. The aminoquinolinium salts did presumably not interfere with actin ADP-ribosylation or receptor binding but blocked the pores formed by C2IIa and Iota b in living cells and in vitro. The blocking efficiency of pores formed by Iota b and C2IIa by the chloroquine analogs showed interesting differences indicating structural variations between the types of protein-conducting nanochannels formed by Iota b and C2IIa.

## 1. Introduction

Binary A-B type protein toxins are potent virulence factors of certain gram-positive bacteria (for reviews see refs [1,2,3]). The most prominent example of this type of toxins is the anthrax toxin produced by *Bacillus anthracis*, which is also known as a possible biological weapon [4,5,6]. Other prominent examples are C2-toxin of *Clostridium botulinum* and Iota-toxin of *Clostridium perfringens*. Both toxins consist of two distinct components that are secreted separately into the extracellular media: an enzymatically active component A—which acts as an actin-specific ADP-ribosyltransferase—and a separate component B, which is the binding/translocation subunit needed for binding of the toxins to target cells and responsible for translocation of the enzymatic subunits into the cytosol of target cells [4,7,8,9,10,11,12].

After the proteolytic activation the B components C2II of *Clostridium botulinum* and also Iota b of *Clostridium perfringens* form ring-shaped heptamers similar to the B component of the anthrax toxin PA [11,13,14,15,16]. These heptamers (C2IIa, Iota b) are the biologically active species of the B components and mediate two different functions during cellular uptake of the toxins: First, they bind to their receptors on the surface of target cells and form complexes with their A components. These complexes are subsequently taken up into cells via receptor-mediated endocytosis and thereby reach early endosomal vesicles. The acidic conditions in such endosomes trigger a conformational change of the compound B heptamers, which insert into endosomal membranes to form trans-membrane pores. These pores serve as translocation channels for the subsequent transport of the unfolded A components of these toxins from the endosomal lumen into the host cell cytosol. Treatment of cells with bafilomycin (Baf) A1, a compound that prevents acidification of the endosomes, inhibits pore-formation by the B components, and therefore the translocation of the A components across endosomal membranes into the cytosol and thus protects cells from intoxication with these toxins [1,17,18,19,20]. Such a translocation mechanism is common to other binary toxins, including anthrax toxin from *Bacillus anthracis* [1,21].

The enzymatic components develop their activity in the cytosol of the target cells where they ADP-ribosylate monomeric G-actin at position arginine 177 with NAD as co-substrate leading to actin depolymerization, cell rounding, and eventually cell death [1,22,23,24,25,26]. Similarly, other members of the family of binary toxins act also as ADP-ribosylating toxins. These are CDT (*Clostridium difficile* binary toxin) of *Clostridium difficile* [27,28,29], *Clostridium spiroforme* toxin [30], and the vegetative insecticidal proteins (VIPs) of *Bacillus cere*us and *Bacillus thuringiensis* [31,32].

The inhibition of channel function by binding components and intoxication of target cells by compounds that bind to the binding components is of considerable interest because of the possible use of A-B type of toxins as biological weapons. Possible candidates are tailored azolopyridinium salts and tailored cyclic dextrines [33,34,35,36]. In previous studies, we have demonstrated that low concentrations of chloroquine were able to inhibit intoxication of target cells by C2-toxin in cell-based assays and pore-formation by C2IIa in lipid bilayer membranes [37,38]. Similarly, blockage of iota b channels by chloroquine was also observed in reconstitution experiments with lipid bilayers but at much higher concentrations than those needed in experiments with C2IIa [39,40]. The binding site for chloroquine and related compounds in the channel formed by C2IIa was identified in the vestibule on the cis-side of the mushroom-sized heptamers that corresponds to the cell surface exposed side [41]. It is presumably the same binding site that also interacts also with the positively charged N-terminus of the enzymatic subunits C2I and Iota b and directs them to the channel lumen and further on into the cytosol of the target cells [1,3,40]. This means that binding is the prerequisite for transport. Site-directed mutagenesis of E399, D426, and F428 (corresponding to the Φ–clamp in PA [42,43]) in C2IIa has clearly demonstrated that these three amino acids are elements of the binding site within the vestibule of the channel formed by C2II [41]. These amino acids are also present in the primary sequence of Iota b in similar positions (D386, D413, and F415) and there exists no doubt that they are also involved in the binding site of the heptameric Iota b channel [40]. Besides these amino acids that are directly involved in binding of Iota a and chloroquine the sequence of Iota b also contains several threonines (T292 and T320) that are probably involved in the structure and stability of the pore-forming heptamers of Iota b. Their replacement by other amino acids leads to misfolded Iota b channels that have completely different properties than the ones formed by wildtype Iota b [40].

In this study, we investigated the binding of different chloroquine analogs to the channels formed by the binding components C2IIa and Iota b. The interaction between the protein-conducting nanopores and the different ligands was performed by titration experiments with artificial membranes containing C2IIa and Iota b channels. This type of investigation using the dose-dependent decrease of membrane conductance allowed a rapid and meaningful investigation of the affinity of the different chloroquine analogs to the binding site inside the vestibule of the heptameric channels. Similarly, we investigated the effect of the chloroquine analog with the highest affinity for binding to C2IIa and Iota b on the pH-dependent trans-membrane transport of the A components of C2- and Iota-toxin through the trans-membrane pores formed by the B components of these toxins in living cells. The results suggested indeed that this compound blocked the trans-membrane transport of these binary toxins with much higher efficiency than chloroquine.

## 2. Results

### 2.1. Binding of Different Aminoquinolinium Salts to the Channels Formed by the Binding Components C2IIa and Iota b

**Figure 1:**
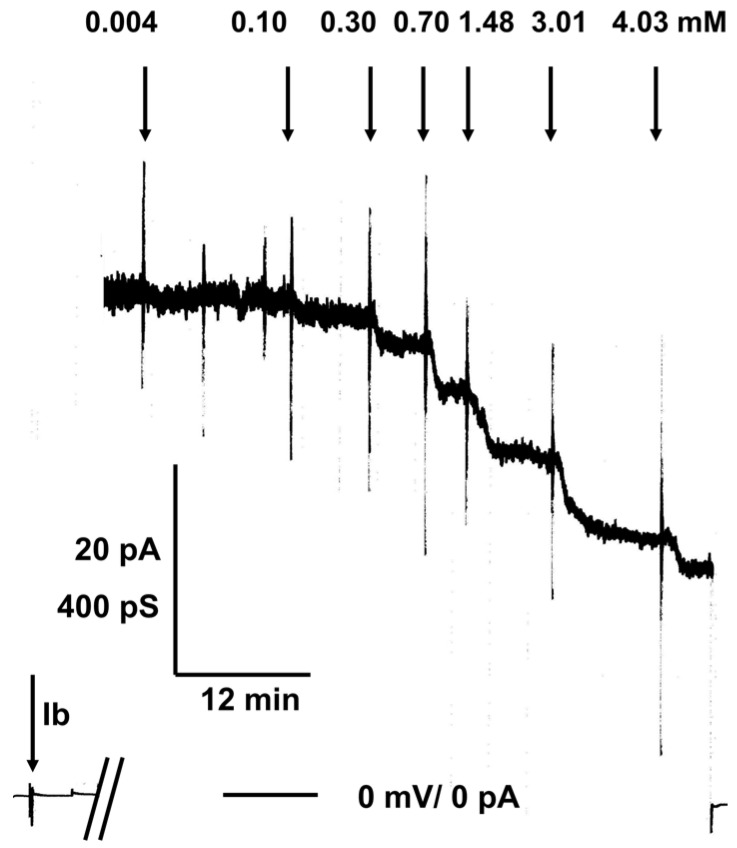
Titration experiment of Iota b-induced membrane conductance with C 164. The membrane was formed from diphytanoyl phosphatidylcholine/n-decane. The aqueous phase contained 20 ng/mL Iota b (Ib) protein (added to the *cis*-side of the membrane at the time of the left side arrow), 150 mM KCl, 10 mM MES, pH 6.0. The temperature was constantly 20 °C and the applied voltage was 50 mV. The two bars at the base line indicate the time interval of about 1h and 50 min between the addition of Ib and the start of the titration experiment. The membrane contained about 70 Iota b-channels (single channel conductance G = 15 pS) when C 164 was added at the indicated concentrations to the aqueous phase. The bottom line represents zero level of conductance (at begin of the experiment) or also zero level of current when voltage was switched off (at the end of titrations). Note that the high noise of the current recording during the titration experiment was caused by stirring in the membrane cell to allow rapid equilibration of C 164 in the aqueous phase.

The channels formed by the binding components C2IIa and Iota b are fully oriented in artificial and presumably also in biological membranes when they are added to only one side of an artificial and biological membrane [37,39]. Most of the water-soluble part of the mushroom-sized heptamer is localized on the *cis*-side of the membrane (the side of addition of the binding components). Only a few amino acids at the end of the beta-barrel cylinder of 14 beta-strands are directed to the trans-side of the membrane. The structure is similar to that of α-toxin of *Staphylococcus aureus* and the recently elucidated 3D-structure of the membrane-spanning form of the PA_63_-channel, which forms also a heptamer with some sort of vestibule on the *cis*-side [16,44]. In previous studies we demonstrated that reconstituted C2IIa channels as well as Iota b channels can be blocked in lipid bilayer membranes by the addition of 4-aminoquinolines [38,39,45] and identified the binding site for chloroquine to C2IIa channels on the *cis* side of the C2IIa heptamer within the vestibule of the channels [41]. The binding affinity strongly depends on negatively charged amino acids and also on the Φ-clamp within the vestibule of the C2IIa and Iota b channels. The stability constant *K* for ligand binding to the C2IIa and the Iota b channels was calculated by multi-channel titration experiments [37,38,39,40]. Similar experiments were performed here with the chloroquine analogs C 23, C 164, C 268, and C 280. Figure 1 shows an experiment of this type. Activated Iota b was added in a concentration of about 20 ng/mL to the *cis*-side (the side of the applied potential) of a black lipid bilayer membrane while stirring. The reconstitution of Iota b channels led to a substantial increase of membrane conductivity by several orders of magnitude caused by insertion of Iota b channels in the membrane monitored by a strip chart recorder. After about 30 min to several hours, when the membrane conductance was virtually stationary, the titration experiments started. Small amounts of concentrated solution of C 164 were added after about two hours after the start of the experiment to the aqueous phase on the *cis*-side of the membrane while stirring to allow equilibration. Subsequently, the Iota b channels were blocked and the dose-dependent decrease of conductance was measured as a function of time (see Figure 1).

**Figure 2:**
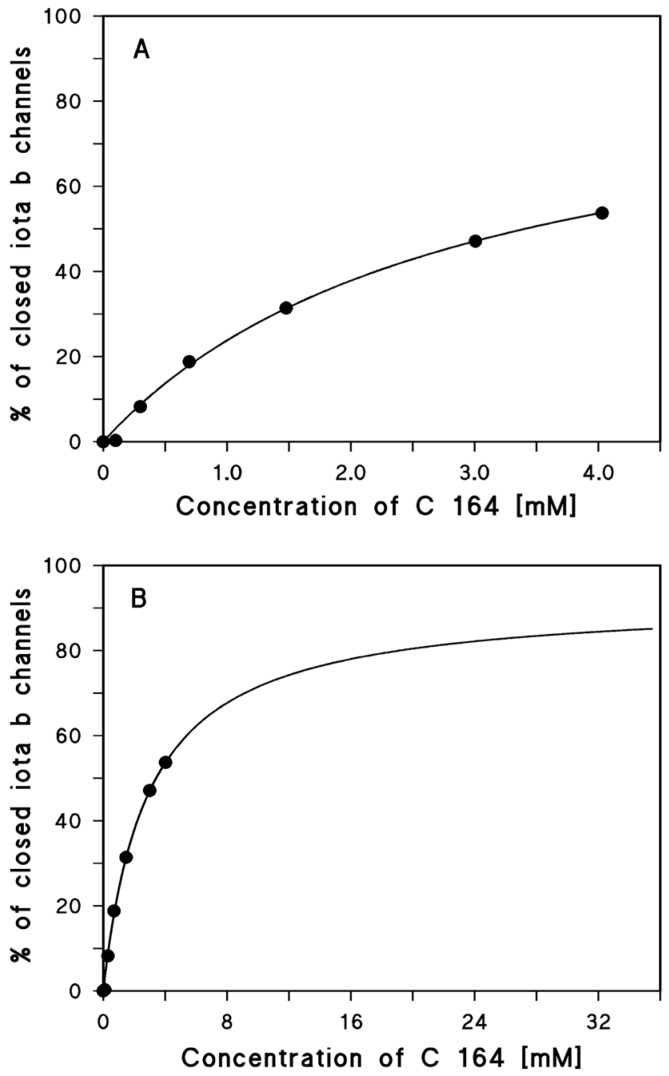
(**A**) Langmuir isotherm of the inhibition of Iota b-induced membrane conductance (about 70 Iota b-channels) by the aminoquinolinium salt C 164. The fit line corresponds to the data points taken from the titration experiment in Figure 1. The fit of the data was performed using Equation (2). The stability constant, *K*, for binding of C 164 to the Iota b-channels was (348 ± 48) 1/M (The channel block was at maximum 92% ± 7%; *K_S_* = 2.9 mM (*r*^2^ = 0.997645)); (**B**) Because of the low degree of inhibition in C 164 concentration range, we extrapolated its concentration to about 33 mM and used the same fit parameters as in A. The fit curve indicates that high concentration of C 164 almost fully blocked the Iota b channels.

**Figure 3:**
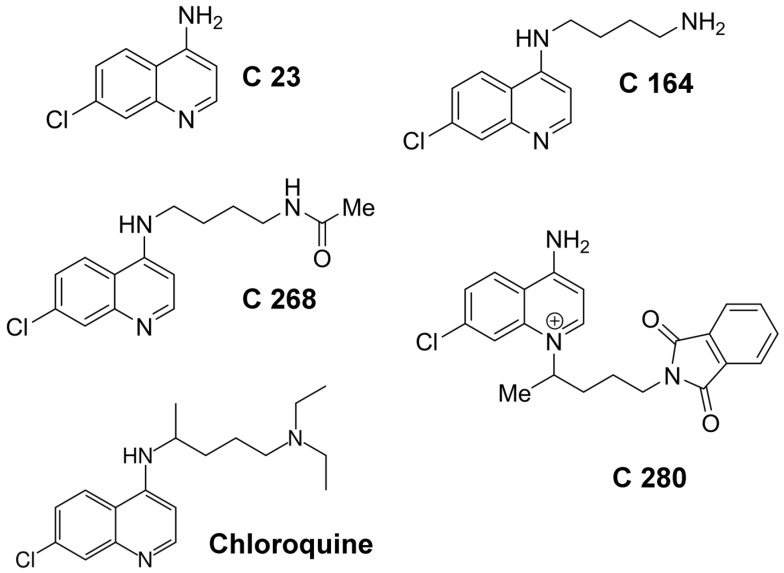
Structures of chloroquine and the chloroquine analogs (aminoquinolinium salts) used in this study). The chloroquine analogs were designated as suggested by Lödige (2013) [46].

The analysis of the data of Figure 1 indicated that the Iota b channels were not fully blocked by the addition of C 164 at a concentration of 4.03 mM. This was caused by the problem to reach sufficiently high concentrations of the chloroquine analog, which are limited by its solubility in aqueous salt solution. The fit of the titration data shown in Figure 2A suggest in principle that the Iota b channel was only blocked by about 50%. However, when the concentration of C 164 was extrapolated to higher ones (see Figure 2B), then it was clear that compound C 164 was also able to almost fully block the Iota b channel. The stability constant of binding of C 164 to the Iota b channels was about (348 ± 48) 1/M and the channel block was at maximum 92% ± 7%. This was a very low stability constant for binding of a channel blocker to one of the binding component channels. However, the data of binding of all chloroquine analogs and chloroquine itself demonstrates that the iota b channel is not a good target for binding of chloroquine and the different chloroquine analogs (aminoquinolinium salts) (see Table 1). Only chloroquine itself and C 280 had a reasonably high affinity to the Iota b channel. Chloroquine analog C 280 that has a permanent positive charge and a bulky side chain was used for the study of the inhibition of cell cytotoxicity by Iota-toxin (see Figure 3).

The affinity of chloroquine and the chloroquine analogs to the channels formed by C2IIa was definitely more substantial as the summary of stability constants and half saturation constants of Table 1 clearly demonstrates. Chloroquine and all aminoquinolinium salts used in this study have a higher affinity to the C2IIa channel than to that formed by Iota b. With the aminoquinolinium salts, the affinity to the C2IIa channels increased in the series C 23, C 164, C 268, and C 280 by factor of more than 4000. C 268 had already a binding constant to C2IIa channels that was about twofold higher than chloroquine. The highest stability constant for binding to the C2IIa channel was C 280, which had a half saturation constant of 0.16 µM. This low *K_S_* value for binding of C 280 to C2IIa is about a factor of 60 smaller than that of chloroquine binding which is remarkable and suggested indeed that C 280 could serve as an inhibitor of intoxication by C2-toxin.

### 2.2. C 280 Inhibited pH-Dependent Membrane Translocation of C2 and Iota Toxin

Prompted by the observation that C 280 interfered with the C2IIa and Iota b pores in vitro, we addressed the question whether C 280 also inhibits translocation of the enzyme components C2I and Iota a through their respective toxin pores across the membranes of intact cells. To this end, we used an established assay, which mimics the acidic conditions of endosomes on the surface of intact cells and allows direct translocation of the C2I and Iota a enzyme components into the cytosol through CIIa and Iota b pores, respectively, which were inserted in the plasma membrane under acidic conditions [34]. All steps of this assay were performed in the presence of Baf A1 to block the normal uptake of C2 and Iota toxins into the cytosol via acidified endosomes.

**Figure 4:**
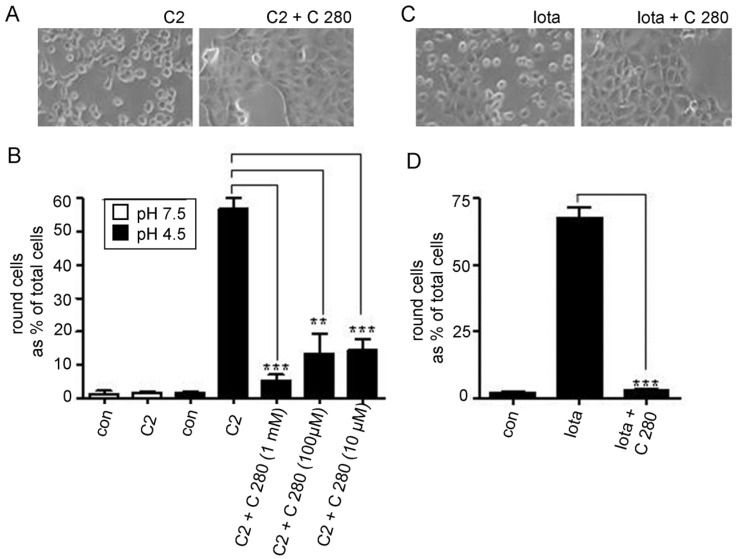
Inhibitory effect of C 280 on the pH-dependent trans-membrane transport of the C2 and iota toxins in living cells. (**A**) Baf A1-treated Vero cells were incubated for 30 min at 4 °C with C2 toxin (100 ng/mL C2I + 200 ng/mL C2IIa) to enable toxin binding. Noteworthy, Baf A1 was present to inhibit in a later step the “normal” transport of the A components of the internalized toxins into the cytosol via acidified endosomes, which is a prerequisite to investigate the toxin transport across the cytoplasmic membrane in this approach. For control (con), cells were incubated without toxin. Subsequently, cells were exposed for 5 min at 37 °C to acidic medium (pH 4.5 or to neutral medium pH 7.5 for control) and subsequently incubated at 37 °C in neutral medium containing Baf A1. During the acidic pulse, the B components insert as pores into the cytoplasmic membrane and the A components translocate through these pores into the cytosol of the cells and induce ADP-ribosylation of actin and cell-rounding. In this approach, the toxin-induced cell-rounding serves as an established specific and sensitive endpoint to monitor the uptake of the A components into the cytosol in the presence and absence of the inhibitor. To test the effect of C 280 on toxin translocation, the indicated concentrations of C 280 were present in the medium during acidic pulse and the subsequent incubation periods. Pictures were taken after 1 and 2 h to document cell rounding, i.e., intoxication of cells (shown in A for 2 h and 1 mM C 280); (**B**) The percentage of intoxicated cells was determined after 1 h, and values are given as mean ± S.D. (*n* = 3). Significance was tested between cells, which have been treated with C2 toxin either in the absence or presence of C 280 by using the student’s *t*-test (*** *p* < 0.0005, ** *p* < 0.005); (**C**) To test the influence of C 280 on membrane translocation of iota toxin, Baf A1-treated Vero cells were exposed for 15 min at ph 4.0 to Iota toxin (100 ng/mL Ia + 200 ng/mL Ib) in the presence or absence of 1 mM C 280. Cells were incubated for further 2 h at 37 °C in neutral medium containing Baf A1 in the presence or absence of C 280. Pictures were taken and the percentage of round cells was determined (D) Values are given as mean ± S.D. (*n* = 3). Significance was tested between cells, which have been treated with iota toxin either in the absence or presence of C 280 by using the student’s t-test (*** *p* < 0.0005, ** *p* < 0.005).

Vero cells were exposed to either C2 or Iota toxin under acidic conditions in the presence and absence of C 280 and subsequently the cells were further incubated under neutral conditions also with or without C 280. As shown in Figure 4, C2- and Iota-treated cells rounded up consequently to the acidic pulse. However, significantly less cells were rounded in the presence of C 280, indicating that less C2I or Iota a reached the cytosol. This result strongly suggests that C 280 inhibits membrane translocation of C2I and Iota a through the lumen of the C2IIa and Iota b pores, respectively. This result suggested that chloroquine-analogs similar to the structure of C 280 might provide interesting tools for the further study of aminoquinolinium salts as blockers for intoxication by C2-toxin. The block of intoxication of Iota-toxin by the same molecules does not look so promising because of the possible different structure of the Iota b channel (see Discussion).

## 3. Discussion

### 3.1. The Structure of the Aminoquinolinium Salts Allows an Interesting Insight in Structural Elements Required for Efficient Binding Protein Channel Blocking

The experiments with different aminoquinolinium salts to block the C2IIa channel allow an interesting insight in the structural requirement for efficient channel blocking, which can be considered as important tool for further in vivo and in vitro studies. The simplest chloroquine analog in the study here is C 23 (4-amino-7-chloroquinoline) which represents only the heterocyclic (bicyclic) part of the chloroquine molecule without side chain. C 23 has the smallest binding affinity to the C2IIa channels with a half saturation constant of about 700 µM for binding to C2IIa. The addition of n-butylamine to the amino group at the bicyclic molecule C 23 decreased the half saturation constant *K_S_* for binding to C2IIa by a factor of more than 10 to 54 µM. The further addition of an acetyl group to the amino group of C 164 led to an additional decrease of the half saturation constant for the resulting C 268 molecule to about 5 µM. The half saturation constant is already below that of chloroquine, which means that C 268 is already a more efficient blocker of C2IIa channels than chloroquine. Another big step to improve binding of the aminoquinolinium salts to the C2IIa channel is the attachment of the bulky side chain to the nitrogen in the pyridine ring of C 23 (see Figure 3). The resulting chloroquine analog (C 280) has the highest affinity for binding to C2IIa but also for binding to Iota b. The half saturation constant for C 280 binding to C2IIa drops down to 0.16 µM, which is about 60-times lower than the half saturation constant for chloroquine binding.

The situation is in the case of binding of the aminoquinolinium salts to the Iota b channel not such straightforward as in the case of C2IIa. The reason for this is that the half saturation constant for binding shows some increase from C 23 to C 164 and then starts to become smaller in the series C 164, C 268, and C 280 (see Table 1). However, all half saturation constants for binding of the aminoquinolinium salts to Iota b were considerably higher than those for their binding to C2IIa. Even C 280, which had the highest binding affinity to Iota b with a half saturation constant of 80 µM showed a half saturation constant that was about 500 times higher than the corresponding constant for binding to C2IIa, which is remarkable. Similarly, the half saturation constant for binding of C 280 to the Iota b channel (80 µM) is only little smaller that for binding of chloroquine to the channel (140 µM), which looks a little strange when the situation is again compared to that of C2IIa. Nevertheless, the binding of C 280 to the Iota b channel is still strong enough that inhibition of the translocation of Iota a through Iota b is blocked in cell-based assays. This means that also certain aminoquinolinium salts could serve as blockers for intoxication of cells by Iota-toxin.

### 3.2. What Could Be the Reason That Blockers of Channel Function Have a Much Smaller Affinity to Iota b Than to C2IIa?

The results presented here and in previous studies demonstrated that chloroquine had much lower binding affinity to Iota b channels as compared to binding to C2IIa heptamers [37,38,39,40]. Similarly, the affinity of the aminoquinolinium salts to both binding protein channels differed considerably (see Table 1). The reason for this discrepancy is not quite clear because many structural elements that probably contribute to the binding site in the vestibule of the two channels are present in both primary sequences. There are the two rings of—at maximum—seven negatively charged residues in the binding site of the heptamer (D386 in Iota b corresponding to E399 in C2IIa and D413 in Iota b corresponding to D426 in C2IIa [41]). The only difference is that Iota b contains aspartate in position 386 whereas C2IIa has in the corresponding position 399 a glutamate. However, this very conservative exchange should not interfere much with the properties of the binding site in the vestibule of the two heptamers. Similarly, both cannels contain an Φ-clamp (F415 in Iota b and F428 in C2IIa), which is also an important structural element in both channels [40,41]. Thus, it is only slightly understandable that they differ so substantially in binding of the aminoquinolinium salts. The only remarkable difference between Iota b and C2IIa is the number of negatively charged groups within the channel-forming domain itself [41]. Whereas the membrane-spanning beta-sheet structure of C2IIa contains one glutamate (E307), there are no charges in the membrane spanning part of Iota b. This could represent the difference. However, experiments with a C2IIa mutant where E307 was replaced by lysine demonstrated that the E307K C2IIa mutant channel had approximately the same affinity for chloroquine as wildtype C2IIa [47]. This means presumably that charges within the pore-forming domain of the binding protein heptamers are most likely not essential for aminoquinolinium salt binding. This has to do with the strong image force along the channel that is created by the many negatively charged groups within the vestibule of the channels [47]. Taken together, it seems moreover that the structure of the binding site within the vestibule of the Iota b heptamers shows some structural differences to those of C2IIa and PA that result in a lower affinity for binding of chloroquine and its analogs [41,48].

### 3.3. The Aminoquinolinium Salts Inhibit the Trans-Membrane Transport of the A Components of the Binary C2 and Iota Toxins Through the Pores Formed under Acidic Conditions by the B Components in Membranes of Living Cells and Protect Cells from Intoxication with These Toxins

As expected from the in vitro results with lipid bilayers, the compounds inhibited the pH-triggered trans-membrane transport of the enzyme components of C2-toxin and Iota-toxin through the pores formed by the B-components of these toxins in cell membranes under acidic conditions, thereby protecting the cells from intoxication. This was tested in a well-established experimental approach where the situation of acidic endosomal vesicles was experimentally mimicked on the surface of living cultured epithelial cells. Vero cells were pre-treated with Baf A1 to inhibit endosomal acidification and thereby prevent the “normal” uptake of the binary C2 and iota toxins via acidic endosomes [17,18,19]. Then, the cells were incubated for 30 min at 4 °C with the respective toxin to enable toxin binding to the cell surface receptors but not receptor-mediated endocytosis, which does not occur at 4 °C. Subsequently, the cells were exposed to a short acidic pulse at 37 °C to trigger the conformational change of the cell-bound B components, which then form pores in the cytoplasmic membrane and mediate the transport of their bound A components through these pores across the cytoplasmic membrane into the cytosol of the cells. This transport of the A components into the cytosol results in ADP-ribosylation of actin and cell-rounding, which serves as a highly specific and sensitive endpoint to monitor the uptake of the A components into the cytosol in the presence and absence of the inhibitor. No cell-rounding was observed without toxin, indicating that the acidic conditions alone had no effect on cell morphology, or when cells were exposed to neutral medium because in this case, Baf A1 prevented the toxin uptake into the cytosol (Figure 4). As shown in Figure 4, cells only rounded up when the toxin was bound prior to the acidic pulse, indicating translocation of the A components into the cytosol. Significantly fewer cells rounded up under such conditions in the presence of C 280, clearly indicating the inhibitory effect of this compound on the membrane transport of the binary toxins C2 and iota in living cells. The aminoquinolinium salts had no effect on cell morphology under the experimental conditions used in this study. They have also the unique advantage that their toxicology is known from malaria treatment. In conclusion, these compounds should represent attractive lead compounds for development of novel pharmacological inhibitors against binary clostridial actin ADP-ribosylating toxins and may further be used against related binary toxins from pathogenic bacteria.

## 4. Experimental Procedures

### 4.1. Materials

The recombinant components of C2 toxin, C2I and C2II, were expressed as GST fusion proteins in *Escherichia (E.) coli* BL21 cells and purified as described [8]. To obtain biologically active C2IIa, C2II was treated with trypsin as reported earlier [8]. Iota a and Iota b were kind gifts of Dr. Michel R. Popoff (Institut Pasteur, Paris, France) [11]. Iota a and Iota b were activated by α-chymotrypsin as described previously [11,40,49]. The heterocyclic chloroquine analogs (aminoquinolinium salts) C 23, C 164, C 268, and C 280 (see Figure 3) were kind gifts of Dr. Gerhard Bringmann and Dr. Melanie Lödige, Institute for Organic Chemistry, University of Würzburg, 97074 Würzburg, Germany. The chloroquine analogs were termed and synthesized as was described in detail recently [46]. The chloroquine analogs were dissolved in ultrapure water supplemented with 10% (*v/v*) ETOH. Cell culture media (DMEM, MEM) and fetal calf serum were obtained from Invitrogen (Karlsruhe, Germany) and cell culture materials from Techno Plastic Products. (Trasadingen, Switzerland). Complete^®^ protease inhibitor and streptavidin-peroxidase were from Roche (Mannheim, Germany), Baf A1 from Calbiochem (Bad Soden, Germany), and biotinylated NAD^+^ from R&D Systems GmbH (Wiesbaden-Nordenstadt, Germany).

All salts (analytical grade) were obtained from Sigma-Aldrich Chemie GmbH (München, Germany) and were dissolved in ultrapure H_2_O (Milli-Q^®^ systems, Merck Millipore, Darmstadt, Germany). Diphytanoyl phosphatidylcholine (DiPhPC) was obtained from Avanti Polar Lipids Alabaster AL and n-decane (analytical grade from Merck, Darmstadt, Germany).

### 4.2. Methods

#### 4.2.1. Cell Culture and Cytotoxicity Tests

African green monkey kidney (Vero) cells were cultivated at 37 °C and 5% CO_2_ in MEM containing 10% FCS, 1.5 g/L sodium bicarbonate, 1 mM sodium-pyruvate, 2 mM l-glutamine, 0.1 mM non-essential amino acids. Vero cells were reseeded twice a week for, at most, 15–20 times. The macrophage-like murine J774A.1 cells were cultivated at 37 °C and 5% CO_2_ in DMEM containing 10% FCS and 4 mM l-glutamate. For cytotoxicity experiments with C2-toxin or Iota-toxin, Vero cells were incubated at 37 °C in 1 mL serum-free medium containing both components of C2 toxin (200 ng/mL C2IIa + 100 ng/mL C2I) or iota toxin (200 ng/mL Iota b + 100 ng/mL Iota a). After different incubation periods, the toxin-induced cell-rounding was documented with a Zeiss Axiovert 40CFl microscope (Zeiss, Oberkochen, Germany) containing a Jenoptik progress C10 CCD camera (Carl Zeiss GmbH, Jena, Germany) and the percentage of round cells was determined from the pictures [19]. Inhibitory effects of C 280 were analyzed by incubating the cells with toxin in the presence of C 280.

#### 4.2.2. Toxin-translocation Assay with Intact Vero Cells

The pH-dependent translocation of C2 toxin across the cytoplasmic membranes of intact Vero cells was performed as described earlier [8]. In brief, Vero cells were pretreated for 30 min at 37 °C with Baf A1 (100 nM) to prevent normal internalization of the toxin via acidified endosomes. Subsequently, cells were incubated at 4 °C in serum-free medium with C2IIa (200 ng/mL) and C2I (100 ng/mL) to enable toxin binding. Cells were washed and exposed for 5 min to warm acidic medium (37 °C, pH 4.5, Baf A1) to trigger insertion of cell-bound C2IIa into the cytoplasmic membrane and subsequent translocation of C2I through the pores across the membrane. Subsequently, the cells were further incubated at 37 °C in complete medium under neutral conditions in the presence of Baf A1 and C2I-toxin induced cell-rounding was documented by photography. The pH-driven translocation of cell-bound Iota toxin across the cytoplasmic membrane of Vero cells was performed as described earlier by Blöcker et al. (2001) [50]. Baf A1-treated cells were exposed for 15 min at 37 °C to acidic medium (pH 4.0) containing Iota toxin (100 ng/mL Iota a + 200 ng/mL Iota b) and subsequently incubated at 37 °C in neutral medium containing Baf A1. The number of round cells was determined to document the cytopathic action of Iota toxin. To test an inhibitory effect of C 280 on membrane translocation of C2- and Iota-toxins, C 280 was applied to the medium during the acidic pulse and the subsequent incubation periods, pictures from the cells were taken and the number of round cells was determined from the pictures. 

#### 4.2.3. Lipid Bilayer Experiments

The experiments with planar lipid bilayers were performed as has been described previously in detail [51]. In brief membranes were formed by the painting method using DiPhPC dissolved to 1% (*w/v*) in n-decane. The membrane hole had an area of about 0.4 mm^2^ in the thin wall separating two 5 mL compartments in a Teflon cell. The different binding components (C2IIa and Iota b) were added from concentrated solutions to the aqueous phase either immediately before membrane formation or after the membranes had turned black in concentrations of about 1 to 10 ng/mL. The temperature was maintained at 20 °C during all experiments. The membrane conductance induced by channels formed by the binding components C2IIa and Iota b was measured after application of fixed membrane potentials with a pair of silver/silver chloride electrodes with salt bridges inserted into the aqueous compartments on both sides of the DiPhPC membranes. The electrodes were connected in series to a voltage source and a homemade current-to-voltage converter made with a Burr Brown operational amplifier. The amplified signal was monitored on a digital storage oscilloscope (OWON) and recorded on a strip chart recorder.

#### 4.2.4. Titration Experiments with the Different Aminoquinolinium Salts

The binding of the compounds C 23, C 164, C 268, and C 280 (see Figure 3) to the channels formed by the binding components was investigated with titration experiments similar to those used previously to study the binding of carbohydrates to the LamB-channel of Escherichia coli [52] and the binding of tailored azolopyridinium salts to channels formed by protective antigen (PA) [33] and C2IIa [34]. About 30 min after start of the reconstitution of the binding components into lipid bilayer membranes, their reconstitution rate in the membranes became very small. Then concentrated solutions of different aminoquinolinium salts were added to both sides of the membranes while stirring to allow equilibration. The results of the titration experiments were analyzed in a similar way as has been performed previously for the binding of azolopyridinium salts to channels formed by protective antigen (PA) [33] and C2IIa [34]. The conductance, *G(c)* of the membrane at a given concentration, *c*, of the different aminoquinolinium salts C 23, C 164, C 268, and C 280 relative to the initial conductance, *Gmax* (in the absence of the ligands), was analyzed using the following equation, which corresponds to Langmuir adsorption isotherms [33,34]:

(1) $$\%\;\text{of fraction of blocked channels}=\frac{\left(G_{\max}-G(c)\right)}{G_{\max}}=\frac{100\cdot K\cdot c}{\left(K\cdot c+1\right)}$$

*K* is the stability constant for the binding of aminoquinolinium salts to channels formed by Iota b and C2IIa [33]. The half saturation constant, *K_S_* of this process is given by the inverse stability constant 1/*K*. *K* can be derived from the titration experiments by a fit of the experimental data to Equation (1). We did not observe full channel blockage in all titration experiments. In cases of only partial blockage of the C2IIa and Iota b channels, Equation (1) had to be modified to account for the reduced maximum blockage given by A in percent (the maximum degree A of blockage was in all cases between 85% to 100%):

(2) $$\%\;\text{of fraction of blocked channels}=\frac{\left(G_{\max}-G(c)\right)}{G_{\max}}=\frac{K\cdot c}{\left(K\cdot c+1\right)}$$

## Figures and Tables

**Table 1:** Stability constants *K* for the block of binding components channels formed by C2II and Iota b by chloroquine and related aminoquinolinium salts in lipid bilayer membranes ^a^. * The results of similar titration experiments performed with C2IIa channels and chloroquine are given for comparison.

Chloroquine Analog	*K*/10^3^ M^−1^	*K_S_/*µM	*K*/10^3^ M^−1^	*K_S_*/µM
-	C2II		Iota b	
Chloroquine	110 *	9.1 *	7.1 ± 1.7	140
C 23	1.5 ± 0.4	710	0.82 ± 0.21	1200
C 164	18.5 ± 2.5	54	0.39 ± 0.13	2400
C 268	198 ± 15	5.1	2.5 ± 0.4	400
C 280	6200 ± 40	0.16	12.5 ± 2.1	80

^a^ The data represent means ± SD of at least three individual titration experiments. The membranes were formed from diphytanoyl phosphatidylcholine/n-decane. The aqueous phase contained 150 mM KCl, 10 mM MES-KOH, pH 6, and about 10 ng/mL activated C2II or about 20 ng/mL Iota b; T = 20 °C. * The stability constant *K* for binding of chloroquine to C2IIa channels is given for comparison and was taken from Bachmeyer et al. (2003) [45].
